# Imaging characteristics of pleural tumours

**DOI:** 10.1007/s13244-015-0441-x

**Published:** 2015-10-16

**Authors:** Luca De Paoli, Emilio Quaia, Gabriele Poillucci, Antonio Gennari, Maria Assunta Cova

**Affiliations:** U.C.O. di Radiologia, Dipartimento di Scienze Mediche, Chirurgiche e della Salute, Università degli Studi di Trieste, Azienda Ospedaliero – Universitaria AOUTS, Trieste, Italy; Ospedale di Cattinara, Strada di Fiume 447, 34149 Trieste, Italy

**Keywords:** Pleural neoplasm, Malignant mesothelioma, Synovial sarcoma, Solitary fibrous tumour, Primary effusion lymphoma

## Abstract

**Abstract:**

Malignant mesothelioma is doubtless the more known pleural tumour. However, according to the morphology code of the International Classification of Diseases for Oncology (ICD-O), there are several histological types of pleural neoplasms, divided into mesothelial, mesenchymal and lymphoproliferative tumours, that may be misdiagnosed. In this paper we summarise and illustrate the incidence aspects and the clinical, pathological and radiological features of these neoplasms.

***Teaching Points*:**

• *According to the ICD-O, there are 11 different histological types of pleural neoplasm.*

• *Imaging, clinical and histopathological aspects of these neoplasms may be overlapping.*

• *Knowledge of different pleural tumours plays an important role for diagnosis orientation.*

## Introduction

### Pleural anatomy

**Fig. 1:**
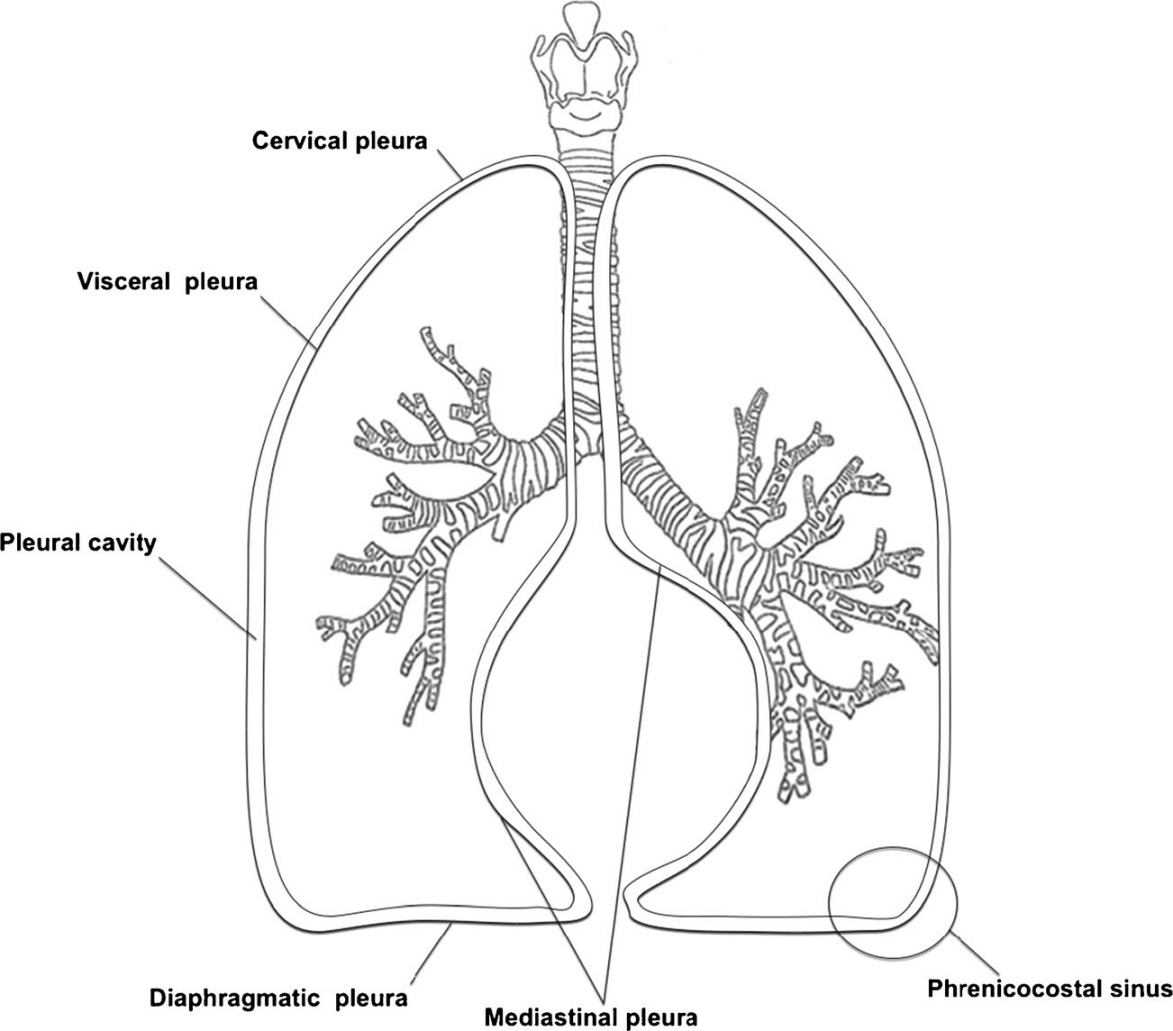
Schematic diagram of the pleurae and upper airways

The two lungs and the pleurae are located in the chest. The pleurae are thin, shimmering serous membranes. From a histological point of view, each pleura is composed of a monolayer of mesothelial cells. These mesothelial cells rest on a matrix of collagen, elastic fibres, blood vessels and lymphatics, allowing the lung and chest to expand and contract [1]. With a rich blood supply and lymphatic system just deep to the mesothelial layer, the pleura forms a dynamic structure protecting the lung from infection while transmitting the forces of respiration without damage to the underlying lung parenchyma. The pleura consists in two sheets: one that covers the thoracic wall and diaphragm, known as the parietal pleura (Fig. 1) and the other called the visceral pleura covering lungs and fissures. Surfaces of the two sheets slide smoothly against each other during respiration. The contact between the parietal and visceral pleurae depends on the atmospheric pressure on the outside of the chest wall and inside the alveoli. Between these layers there is a thin space known as the pleural cavity that contains a small amount of pleural fluid (a few millilitres in a normal human) [2]. The two sheets tend to be separated by the elasticity of the thoracic wall (outward) and the lungs (lengthen by inspiration). Pleura has its own nerves, arteries and lymphatic drainage. The visceral pleura covering the lung itself receive their innervation from the autonomic nervous system and have no sensory innervation. Only the parietal pleurae are sensitive to pain. Parietal pleura consists of three regions: costal, mediastinic and diaphragmatic (Fig. 1). In the anterior region, the mediastinal pleura turns onto the mediastinum and covers the costomediastinal recess. In the lower regions, the costal pleura becomes the diaphragmatic pleura. The anterior borders of the right and left pleurae meet at or close to the median axis determining the origin of mediastinal lines at chest X-ray. At the bottom of the lung, mediastinal pleura goes laterally, circumscribing the anatomical structures at the root and continuing in the visceral pleura. Mediastinal pleura is adherent to the pericardium, except where the phrenic nerve descends between them. On top of the aortic arch, the right and left pleurae approach each other behind the oesophagus. The diaphragmatic pleura covers most of the diaphragm. The diaphragmatic cupola is the continuation of the costal and mediastinal portions of the pleura and gains strength by a thickening of the endothoracic fascia (subpleural membrane), attached to interior part of the first rib and the transverse process of the seventh cervical body.

Malignant pleural mesothelioma (MM) is doubtless the more common pleural tumour, with a characteristic poor prognosis. However, according to the morphology code of the International Classification of Diseases for Oncology, the histotypes of pleural neoplasms can be divided into mesothelial, mesenchymal and lymphoproliferative tumours (summarised in Table 1). It is important to know that there are tumoural histotypes other than MM because of the different behaviour, treatment and prognosis of each neoplasm. For the radiologist, such as for the clinician and sometimes also for the pathologist, it can be very challenging and often impossible to distinguish among the different histotypes of pleural tumours, that may present the same symptoms, signs, histological and radiological aspects. For these reasons, to achieve a precise diagnosis, therapy and prognosis, it is important to consider clinical, radiological, histopathological and immunohistochemical findings.

**Table 1:** WHO histological classification of tumours of the pleura, according to the ICD-O

MESOTHELIAL TUMOURS	LYMPHOPROLIFERATIVE DISORDERS	MESENCHYMAL TUMOURS
1- Diffuse malignant mesothelioma (MM) • Epithelioid mesothelioma • Sarcomatoid mesothelioma • Desmoplastic mesothelioma • Biphasic mesothelioma 2- Localised malignant mesothelioma 3- Well-differentiated papillary mesothelioma 4- Adenomatoid tumour	5- Primary effusion lymphoma 6- Pyothorax-associated lymphoma	7- Epithelioid haemangioendothelioma • Angiosarcoma 8- Synovial sarcoma • Monophasic • Biphasic 9- Solitary fibrous tumour of the pleura 10- Calcifying tumour of the pleura 11- Desmoplastic round cell tumour

## Diffuse malignant mesothelioma

Diffuse MM is a malignant neoplasm arising from mesothelial cells in the pleura, and shows a typical growth along the pleural surfaces; less common is the malignant mesothelioma of the pericardium and peritoneum. It is the most common primary malignancy of the pleura [3].

Up 10,000 mesotheliomas are estimated to occur annually across the population of western Europe, Scandinavia, North America, Japan and Australia [4], while precise registrations are not available in areas that still use asbestos (Eastern Europe, South America, Africa and the rest of Asia, including China. Incidence in Europe ranges by country: in the UK, the nation with the high incidence in the area, the mean annual number of cases is 4.1/100,000 (from 2.9 in Wales to 4.1 in England), while Italy has 2.47/100,000. In comparison, in the US, according to National Cancer Institute records from 1975 to 2010, there is an average of 1.0 new case of asbestos cancer per 100,000 [5].

The most common symptoms of diffuse MM are represented by dyspnoea, usually caused by discreet pleural effusion, chest pain, cough and weight loss. Unusual signs and symptoms of MM include spontaneous pneumothorax, laryngeal nerve palsy and superior vena cava obstruction, due to mediastinal invasion of the neoplasm [6].

From a histopathological point of view there are four main histotypes of diffuse MM, represented by epithelioid, sarcomatoid, desmoplastic and biphasic MM.

Epithelioid MM shows a wide range of morphological patterns, which frequently coexist in the same tumour. A tubular histological architecture may be seen, as a papillary or microglandular histological architecture, with more or less connective tissue. Sometimes psammomatous bodies and keratin are observed, which are useful clues in the differential diagnosis among large cell lymphoma, metastatic malignant melanoma and pleural epithelioid haemangioendothelioma.

Sarcomatoid MM is similar to a fibrosarcoma, but some areas may also resemble osteosarcoma, chondrosarcoma and other sarcomas. For these reasons it can be very difficult to distinguish between sarcomatoid MM and sarcomas or also sarcomatoid-type carcinomas involving the pleura, such as the pleomorphic carcinoma of the lung or the sarcomatoid renal cell carcinoma.

Desmoplastic MM is characterised by dense collagenised tissue with scattered atypical cells, which may be observed in at least 50 % of the tumour, and it may be misdiagnosed even at histology, being confused with benign organising pleurisy.

Biphasic MM is characterised by a combination of sarcomatoid and epithelioid pattern in about 30 % of cases.

MM prognosis is always poor, with a mean survival time of 1 year from the time of diagnosis. Negative prognostic factors are represented by mediastinal lymph node metastasis, thoracic wall and extra-pleural structure involvement, and distant metastases [7]. The histological subtype is also related to prognosis since the epithelioid histotype presents the best survival time, while the sarcomatoid histotype presents the worst prognosis, whereas mixed histotypes show intermediate survival time. According to the 7th AJCC Cancer Staging Manual, all the MM histological histotypes follow the same TNM staging system, as summarised in Tables 2 and 3 [8].

**Table 2:** TNM classification of MM, according to the 7th AJCC Cancer Staging Manual

T (sum of the extent of spread of the primary tumour)	
TX^a^	The main tumour cannot be assessed for some reason
T0^a^	No evidence of a main tumour
T1^a^	MMT involvement of the pleura on one side of the chest (even the diaphragmatic or mediastinal pleura)
T2^a^	T1 + involvement of chest wall, diaphragmatic and mediastinal pleura and at least one of diaphragm or lung infiltration
T3^a^	T2 + infiltration of at least one of the following: endothoracic fascia, fatty mediastinal tissue, a SINGLE place in the deeper layers of the chest wall, surface of the pericardium
T4^b^	T3 + infiltration of at least one of the following: more than one place in the deeper layers of the chest wall, the peritoneum, any organs in the mediastinum, the spine, the other side of the chest, the heart muscle
N (regional lymph nodes involvement)	
NX	The nearby lymph nodes cannot be assessed
N0	No spread to the nearby lymph nodes
N1	Spread to the hilar or bronchial lymph nodes on the same side as the primary tumour
N2	Spread to other lymph nodes on the same side as the primary tumour (subcarinal, mediastinal, internal mammary and peridiaphragmatic)
N3	Spread to the supraclavicular lymph nodes and/or to those on the opposite side to the primary tumour
M (primary tumour spread to the other organs of the body)	
M0	No spread to distant organs or areas
M1	Spread to distant sites (lymph nodes and organs)

^a^MMT may still possibly be surgically removed

^b^MMT has grown too far to be completely surgically removed

**Table 3:** Stage grouping for MM, according to the 7th AJCC Cancer Staging Manual

Stage I	T1, N0, M0
Stage II	T2, N0, M0
Stage III	T1/T2, N1/N2, M0 or T3, N0-N2, M0
Stage IV	T4, any N, M0 or any T, N3, M0 or any T, any N, M1

On chest radiography, MM may appear as unilateral pleural effusion, diffuse or focal pleural thickening or as a real pleural solid lesion. The homolateral lung may be reduced in volume, with elevation of the diaphragm, narrowing of the intercostal spaces and homolateral deviation of the mediastinal axis.

**Fig. 2:**
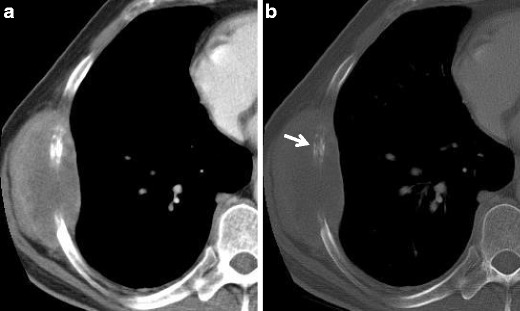
Sarcomatoid mesothelioma; axial view contrast-enhanced CT scan: soft tissue (**a**) and bones (**b**) window images showing a large thoracic wall mass with inhomogeneous enhancement, involving the lateral arch of a rib, that appears eroded (*white arrow*), a typical infiltration sign

Computed tomography (CT) provides more accurate details regarding MM extension, in particular at the level of the chest wall, diaphragm and pericardium, and allows a good evaluation of extra-pleural fat planes, invasion of intercostal muscles and bone erosions (Fig. 2). CT better recognises the pleural thickening, in particular along the fissures that can be so extended to simulate a “rind-like appearance” due to the encasement of the lung [9]. The involvement of mediastinal structures, such as the heart, oesophagus and trachea, or the extra-thoracic spread with involvement of the retroperitoneal space and the liver is more uncommon and typical of extremely advanced cases [6, 10].

Furthermore, CT allows a better evaluation of the hilar and mediastinal lymph nodes and clearly depicts pulmonary metastasis.

**Fig. 3:**
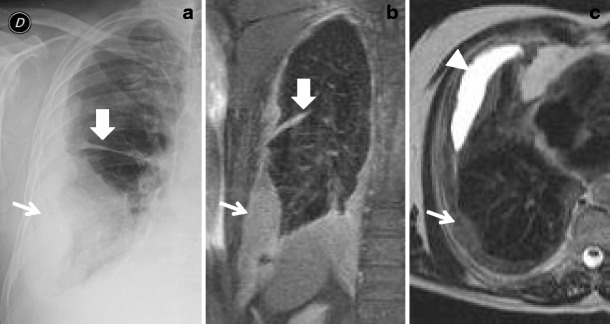
Biphasic mesothelioma; chest X-ray film (**a**) showing a right basal and lateral pleural thickening with fissure involvement (*thick arrow*). Lung MR confirm these findings, both on fat saturation T1-weighted image (**b**) and spin echo T2-weighted sequence (**c**) that better demonstrates a mild basal anterior pleural effusion (*arrowhead*)

CT is essential in MM staging, but it is not very accurate in the evaluation of the early invasion of the structures of the thoracic wall, such as the endothoracic fascia and the intercostal muscles, which leads sometimes to the underestimation of chest wall involvement. Magnetic resonance (MR) imaging, thanks to its high contrast and spatial resolution, allows better assessment of the tumour spread than CT, in particular concerning the invasion of the diaphragm and endothoracic fascia, being very useful in those patients with potentially resectable MM (Fig. 3) [11]. Recent MRI studies have also suggested that the apparent diffusion coefficient (ADC) of the epithelioid subtype of MM is higher than the ADC of the sarcomatoid subtype, a difference that may be a surrogate imaging biomarker [12]. Coolen et al. [13] have suggested the usefulness in diagnosis and guide-for-biopsy of a new MRI sign in diffusion-weighted imaging called ‘pleural pointillism’, defined as multiple (minimum of two) hyperintense areas visible by using a *b* value of 1,000 in the pleura not identifiable with lower *b* values.

Neovascularisation has been shown to be necessary for tumour growth [14] and many studies have confirmed the negative prognosis in highly vascularised MM. Giesel et al. [15] showed that parametric images based on dynamic contrast-enhanced (DCE) MR imaging of MM depicts not only the lesion and its extent but also the heterogeneity in intratumoural vascularisation in MM. The pharmacokinetic parameters may represent predictors of the therapeutic response.

**Fig. 4:**
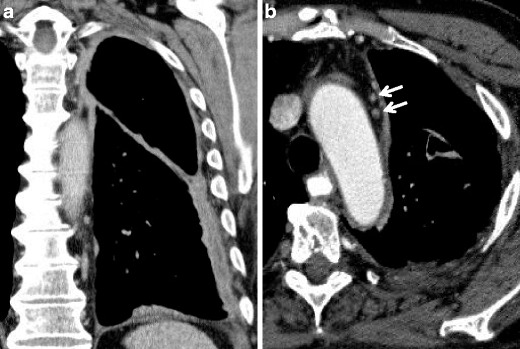
Stage I of MM; contrast-enhanced CT scan: coronal (**a**) and axial (**b**) view. Diffuse circumferential thickening of the thoracic, mediastinal and diaphragmatic pleura involving even the fissure. No extra-pleural infiltration or significant lymphadenopaties are observed (only two small lymph nodes are reported—*white arrows*)

**Fig. 5:**
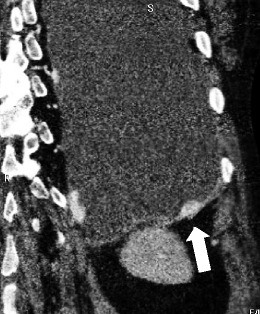
Stage II of MM; contrast-enhanced CT scan: coronal view. Focal pleural enhancing thickening that infiltrates the left hemidiaphragm (*thick arrow*)

**Fig. 6:**
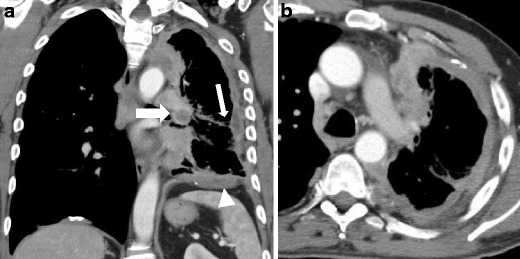
Stage III of MM. Contrast-enhanced CT scan: coronal (**a**) and axial (**b**) view show a severe left lung hypoexpansion, with circumferential irregular pleural thickening and fissure thickening (*white arrow*); left hilar inhomogeneous pathological lymph node may be seen (*thick arrow*), and infiltration of the left hemidiaphragm is reported (*arrowhead*); this MM (sarcomatoid mesothelioma variant) is classified as T2, N1, M0

**Fig. 7:**
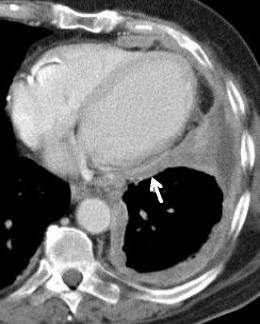
Stage III of MM; contrast-enhanced CT scan: axial view showing a circumferential pleural thickening, involving the mediastinal pleura and the pericardium (*white arrow*), classified as T3, N0, M0

**Fig. 8:**
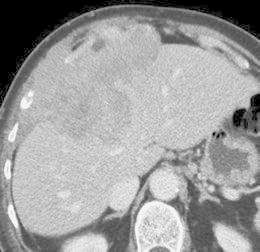
Stage IV of MM; abdominal contrast-enhanced CT scan. Aggressive MM grown through the diaphragm. An axial view demonstrates the infiltration of the peritoneum and the liver (classified as T4)

Positron emission tomography (PET) with 2-[fluorine-18]-fluoro-2-deoxy-D-glucose (FDG) can help in the diagnosis and staging of MM, by using the high standardised uptake value (SUV), which is significantly higher in MM than in benign pleural thickening. In fact, not all the pleural thickenings contain necessarily neoplastic cells, and for this reason PET can guide the chest biopsy, indicating the most FDG-avid tumoural regions. Figures 4, 5, 6, 7 and 8 show some typical aspects of MM staging [16].

Based on the data reported in the literature, this combined modality is also useful in the evaluation of therapeutic outcome and post-treatment surveillance for recurrence, prognosis based upon the intensity of FDG uptake [17].

Ultrasound should also have a role in MM diagnosis. US can be used to guide biopsy to the thickened pleura in MM with pleural effusion. MM presenting without effusion, however, is more of a diagnostic challenge [18]. The therapy is based on surgery (aggressive surgical resection), associated with radiotherapy and chemotherapy, in different protocols [6] However, even with more aggressive and complex therapeutic protocols, the mean survival time hardly reaches 24 months [19, 20].

A relevant topic about MM is the differential diagnosis with pleural plaques, which are relatively common lesions formed by deposits of hyalinised collagen fibres in the parietal pleura. They are indicative of asbestos exposure and typically become visible 20 or more years after the inhalation of asbestos fibres. Differently from MM, pleural plaques usually do not infiltrate adjacent structures and the thoracic wall and are less frequently associated with pleural effusion than MM.

## Synovial sarcoma

Synovial sarcoma is a rare mesenchymal tumour (10 % of all soft tissue sarcomas), usually located in the lower and upper extremities, but in some infrequent cases it arises also within thoracic structures, such as the heart, mediastinum, chest wall, lung and pleura. Synovial sarcoma is thought to originate from primitive (uncommitted) mesenchymal cells that undergo differentiation to resemble synovial cells. This belief is consistent with the malignancy of mesenchymal cells that undergo differentiation to resemble synovial cells [21]

It is more frequent in young patients (average age of 25 years), with males and females affected equally. A precise aetiological factor is not known; however, in all cases a specific chromosomal translocation t(X;18)(p11.2;q11.2) is found [22, 23]. At gross pathology the synovial sarcoma appears as a localised solid tumour, usually arising in the visceral pleura, with very large dimensions (up to 20 cm). Synovial sarcoma often presents cystic areas mixed with necrotic areas, and some of those tumours may also present a pseudocapsule, due to the compression of adjacent compressed lung tissue, with packed blood vessels and granulation tissue [24]

A typical histological feature is represented by a biphasic aspect, with epithelial and spindle cells components, but synovial sarcoma may present also as monophasic neoplasm with exclusively spindle cell component. Both histotypes can be confused with an MM, although synovial sarcoma usually affects younger patients, presents a more localised aspect, grows faster and often presents a pseudocapsule, which is typically absent in the MM [25, 26].

On chest radiography, the chest synovial sarcoma usually appears as a homogeneous round lesion, with well-defined margins, sometimes lobulated, without cavitation, calcification or lymphadenopathy. Calcifications are frequently depicted (up to 30 %) [27].

On CT images, synovial sarcoma appears as a well-defined mass, homogeneous on direct CT, with an irregular enhancement after contrast injection, with some hypodense areas, corresponding with necrotic or haemorrhagic spots. In most cases, a thin peripheral rim of enhancement, corresponding to the pseudocapsule, may be identified. Sometimes consensual pleural effusion is also reported. It is also very hard to distinguish between a primary thoracic synovial sarcoma and a synovial sarcoma metastatic to the pleura, because both forms have almost the same features.

**Fig. 9:**
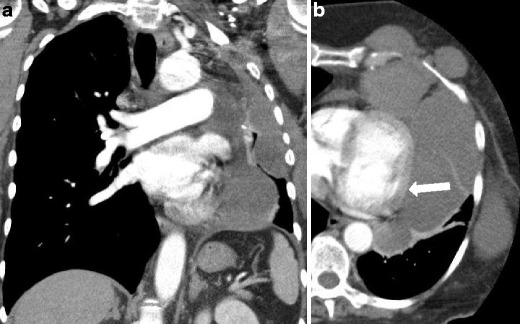
Large left thoracic wall sarcoma; contrast-enhanced CT scan: coronal (**a**) and axial (**b**) view. Severe left hemithorax hypoexpansion, homolateral hemidiaphragm superelevation and presence of a large polylobulated mass, with faint enhancement and extraparietal extension. No precise fat plane may be seen between the mediastinal pleura and the pericardium (*white arrow*), a finding that is highly suspicious for mediastinal infiltration

The extra-pleural synovial sarcoma often shows a destruction of the cortical bone, intratumoural calcifications and infiltration of the adjacent muscular structures. In primary pleural synovial sarcoma, sclerotic reaction of the ribs adjacent to the tumour is observed, without a real lysis of the cortical bone or invasion of the adjacent chest wall structures (Fig. 9).

MRI can depict tumoural nodular soft tissue and multilocular fluid filled components, T1-weighted images typically show a predominantly heterogeneous multilobulated soft-tissue mass with signal intensity similar to or slightly higher than that of muscle. T2-weighted images show prominent heterogeneity [28], with nodular areas of intermediate signal intensity mixed with hyperintense areas (cystic, necrotic, haemorrhagic or mixoid material) [29]. After intravenous contrast media injection, a prominent heterogeneous enhancement may be identified. The administration of gadolinium-based agents is important also to characterise synovial sarcomas with predominantly cystic characteristics [26].

The therapy is primarily based on surgical resection with chemo and radiation therapy. Mean survival time at 5 years is 50-80 %. Worst prognostic factors are the tumour dimensions (more than 5 cm), a non-radical surgical resection, and the presence of distant metastasis.

## Solitary fibrous tumour

Solitary fibrous tumour is another rare pleural neoplasm, representing less than 5 % of all tumours involving the pleura, with no correlation with asbestos exposure. Solitary fibrous tumour has a mass-like form arising both from the visceral and parietal pleura, and more than 50 % of cases show a vascular pedicle.

From the hystopathological point of view solitary fibrous tumours are characterised by both hypocellular and hypercellular areas, and those areas are sustained by a haemangiopericytoma-like fibrovascular stroma. This feature is common also in other fibrous tumours of other tissues.

Common symptoms are cough, chest pain and dyspnoea, even though many patients are asymptomatic, and the solitary fibrous tumour represents an occasional finding. An uncommon sign is hypertrophic osteoarthropathy. A sporadic but pathognomonic sign is the symptomatic hypoglycaemia, due to the production of an insulin-like growth factor by the tumour [30].

**Fig. 10:**
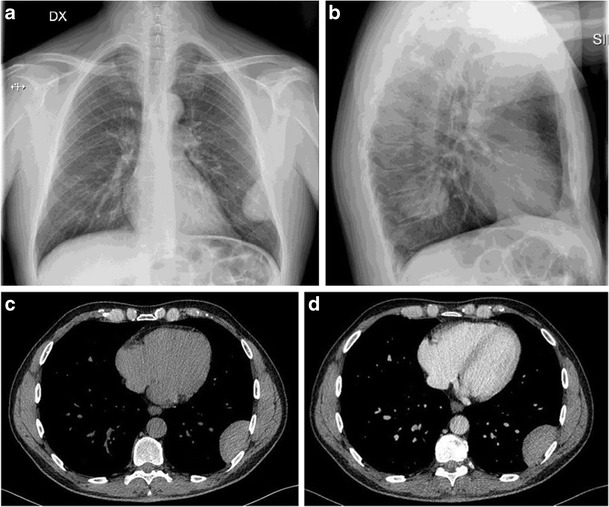
An accidental case of solitary fibrous tumour in a 56-year-old man: standard chest radiography (**a**, **b**) demonstrates a well-defined, ovalar shaped chest wall mass. Contrast-enhanced CT, axial plane before (**c**) and after (**d**) contrast media administration showing a bulky, homogeneus and non-enhancing mass of the left posterior chest wall

On chest radiography a solitary fibrous tumour appears as a homogeneous round mass, with smooth and well-defined margins (Fig. 10). Erosions of adjacent bone structures are extremely rare. Tumours presenting the vascular pedunculus may change in shape and position during breathing and decubitus. Usually the mass forms obtuse angles with the pleural surface on chest radiography, but in the case of large masses, not so infrequent, they form also acute angles. In some cases it can be noticed also a mild homolateral pleural effusion [31].

**Fig. 11:**
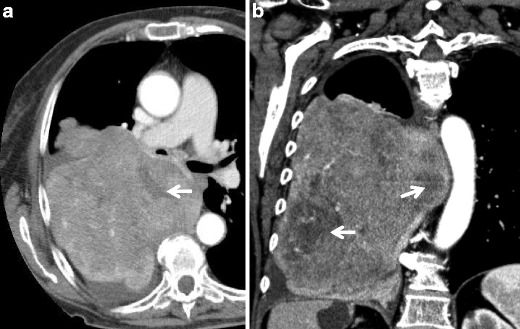
Unusual case of giant solitary fibrous tumour; contrast-enhanced CT, axial (**a**) and coronal plane (**b**) showing a bulky and inhomogeneous contrast-enhancing mass of the right hemithorax. The mediastinal structures are compressed and contralaterally migrated. The hypodense areas (*white arrows*) may represent the presence of necrosis, haemorrhage or myxoid tissue

On unenhanced CT, the solitary fibrous tumour usually appears slightly hypodense, with a slight and homogeneous enhancement after iodinated contrast medium injection in small lesions which may become heterogeneous, in large or giant tumours, due to the presence of necrosis, haemorrhage, mixoid and cystic areas (in 7 % of cases calcifications may be seen). Another peculiarity of solitary fibrous tumour is represented by the compression determined by the large tumoural mass on adjacent lung and mediastinum, rather than by a real infiltration of these structures. Usually there are no locoregional lymphadenopathies (Fig. 11).

The therapy is based on the surgical excision, and the prognosis is good in the majority of patients. However solitary fibrous tumours present a significant incidence of recurrence, and for this reason an aggressive surgical strategy should always be preferred. Malignant solitary fibrous tumours, with a high rate of recurrence and metastasis, have been reported [30].

## Primary effusion lymphoma

Primary effusion lymphoma is a large B-cell lymphoma presenting as an important serous pleural effusion, usually without a real detectable mass. It typically involves the peritoneal, pericardial and pleural cavity.

There is a strong correlation between primary effusion lymphoma and human herpes virus 8 (HHV8)/Kaposi sarcoma herpes virus (KSHV). The virus is often found inside the tumour cells. Usually the patients affected by the primary effusion lymphoma are immunocompromised, in particular HIV positive patients, but also transplanted and elderly patients. In some rare cases the primary effusion lymphoma is associated with multicentric Castelman disease (lymph node benign hamartoma). Both forms are characterised by a high expression of viral interleukin 6 (vIL6), although a sure close connection between the two forms has not yet been established [32]

If primary effusion lymphoma is suspected, a pleural biopsy is necessary. The specimen may show neoplastic cells adherent to the pleural surface, often surrounded by fibrin, and sometimes showing infiltrative aspects. The primary effusion lymphoma must be distinguished from another lymphoproliferative disease of the pleura that is the pyothorax-associated lymphoma. As it will be described subsequently, pyothorax-associated lymphoma usually presents as a pleural mass, positive for Epstein-Barr virus (EBV) and negative for HHV8/KSHV [33].

Typical clinical features of primary effusion lymphoma are the presence of pleural effusion and the absence of lymphadenopathy, and liver or spleen enlargement [34].

**Fig. 12:**
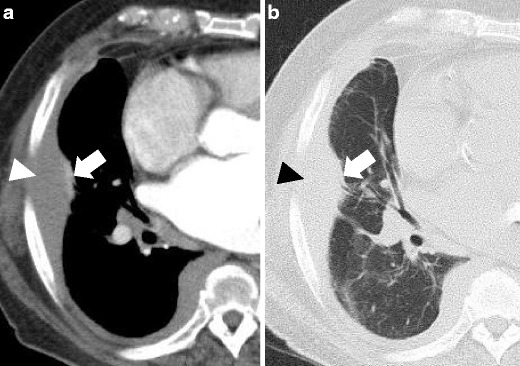
Primary effusion lymphoma; axial view contrast-enhanced CT scan: soft tissue (**a**) and lung (**b**) window images. A mild pleural effusion may be seen (*arrowhead*) with slightly hyperdensity, associated with a small lung consolidation with air bronchogram (*thick arrow*); no real pleural masses are recognised

CT identifies pleural effusion, usually monolateral that may be associated with loss of volume of the involved hemithorax, pleural thickening and the absence of pathological lymph nodes (Fig. 12) [35].

Primary effusion lymphoma is an extremely aggressive disease, with a very poor prognosis (less than a year of mean survival time). The best care is based on several cycles of chemotherapy and antiviral therapy that together seem to show the best prolonged survival.

## Pyothorax-associated lymphoma

Pyothorax-associated lymphoma is another type of large B-cell lymphoma but, unlike primary effusion lymphoma, it presents usually as a real pleural mass. The neoplastic cells are EBV-RNA positive and, as previously said, HHV8/KSHV negative [36].

Usually pyothorax-associated lymphoma arises in patients with a history of chronic pyothorax, typically resulting from iatrogenic pneumothorax for pulmonary tuberculosis or, more rarely, tubercular pleurisy.

Common symptoms are a consequence of the neoplastic mass compression or infiltration of surrounding structures, like chest or back pain, productive cough, fever, dyspnoea, weight loss and dysphagia in case of involvement of the mediastinum. Frequently there is also pleural effusion. In many patients high levels of serum lactate dehydrogenase (LDH) may be observed [37].

On CT, a pleural mass is identified, often with a large dimension showing invasion of the adjacent structures, in particular the thoracic wall, the lung and sometimes the mediastinum. A mild pleural effusion and some pleural calcification may be associated. For this reason it is always important to associate clinical and laboratory data to propose a correct differential diagnosis among pyothorax-associated lymphoma, lung cancer and MM, often manifesting with the same appearance on imaging.

Pyothorax-associated lymphoma presents a very poor prognosis, with a mean survival time of less than 12 months. However, variable protocols of polychemotherapy associated with radiotherapy may lead to higher rate of survival.

## Pleural metastases

**Fig. 13:**
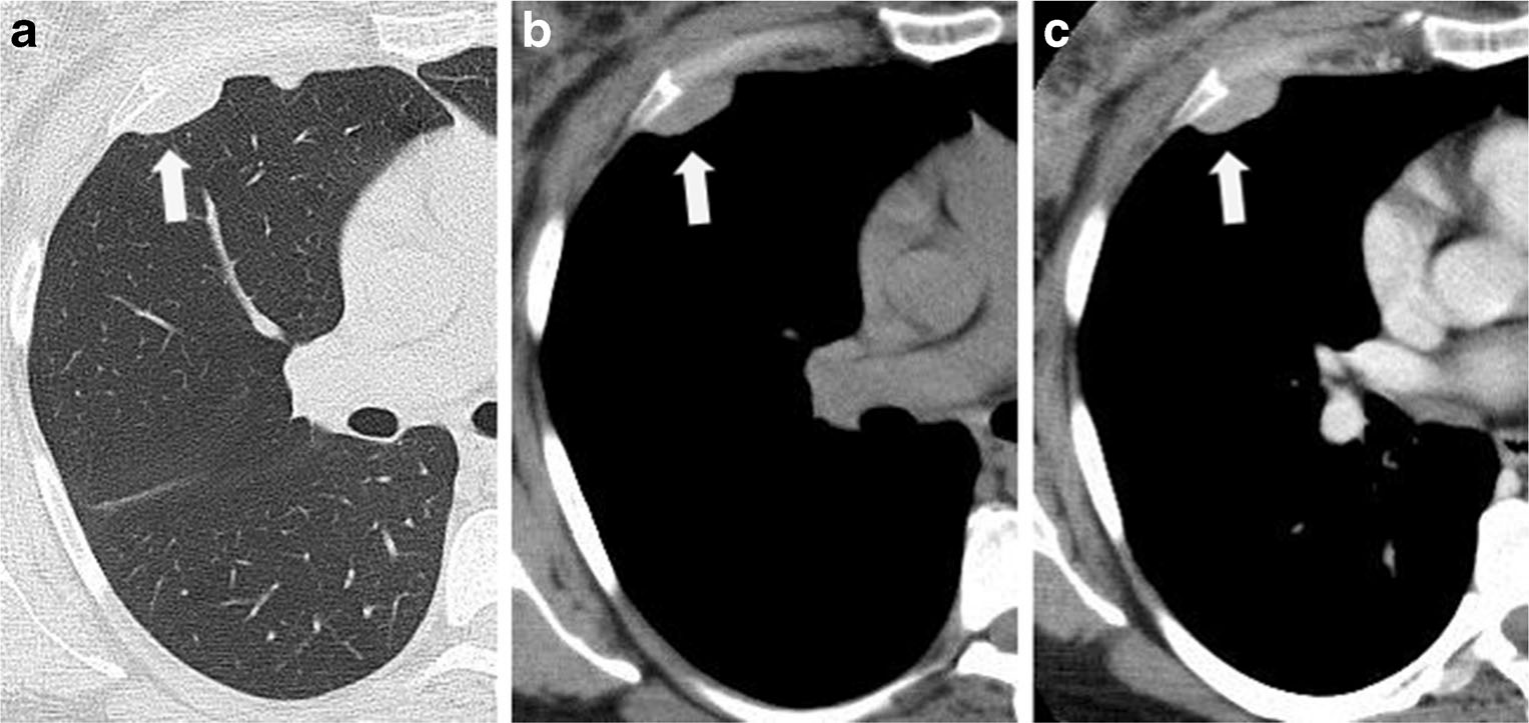
Pleural metastases; axial view of unenhanced and contrast-enhanced CT scan: lung (**a**), soft tissue (**b**) and contrast-enhanced image (**c**). A relatively small bulky mass (*arrow*), that demonstrates slightly and homogeneous enhancement. After analysis of the histological specimen the lesion was found to be a pleural metastases from breast adenocarcinoma

Pleural metastasis (Fig. 13) usually affect both the visceral and parietal pleura. The most common malignancies which metastasise to pleural metastases include: lung carcinoma, accounting for up to 40 % of pleural metastases [38]; breast carcinoma [39], commonly associated with pleural effusion, accounts for nearly 20 % of pleural metastases; ovarian cancers and lymphomas (~10 %).

## Rare pleural tumours

Well-differentiated papillary MM, localised MM, adenomatoid tumour, epithelioid haemangioendothelioma, calcifying tumour of the pleura and desmoplastic small round cell tumour of the pleura represent only a lower percentage of all pleural neoplasms, with not many cases described in the literature.

In all these cases the differential diagnosis among the different histotypes is extremely difficult, especially on small biopsy specimens, mainly because of the similar histopathological appearance and the similar clinical findings. For these reasons a histological and immunohistochemical analysis of the lesions is necessary in order to reach the correct diagnosis.

Localised MM is histologically identical to diffuse MM in all subtypes. Epithelioid haemangioendothelioma and well-differentiated papillary MM may show nests of epithelioid cells, papillary formations and fibromyxoid stroma [40].

Calcifying tumours of the pleura show collagenous and hyalinised tissue with lymphoplasmacytic infiltrates. An adenomatoid tumour is characterised by many eosinophilic cells tending to form tubules and glands. Desmoplastic small round cell tumours of the pleura typically express epithelial, mesenchymal and neural cell markers, and above all a specific translocation t(11;22)(p13;q12).

In well-differentiated papillary MM and localised MM, the correlation with asbestos exposure remains still unclear, with just few reported cases.

These tumours are often asymptomatic, being accidentally discovered during radiological examinations performed for other reasons. Other common symptoms are chest pain, dyspnoea, pleural effusion (more common in well differentiated papillary MM, localised MM, epithelioid haemangioendothelioma and desmoplastic small round cell tumour of the pleura), in relation to their possible malignant behaviour, with invasion of the chest wall, mimicking the typical symptoms of a classic MM.

Well-differentiated papillary MM, adenomatoid tumours and calcifying tumours of the pleura usually present a benign behaviour, and their prognosis is usually good, with a prolonged survival time.

Localised MM, epithelioid haemangioendothelioma, desmoplastic small round cell tumour of the pleura and advanced forms of well differentiated papillary MM show an aggressive behaviour, with both invasion of thoracic wall structures up to the progressive encasement of the adjacent lung parenchyma (frequent in localised MM and epithelioid haemangioendothelioma) and widespread metastasis (more common in desmoplastic small round cell tumour of the pleura). In these cases the prognosis is usually poor, with a mean survival time ranging from months to a few years.

From the radiological point of view, all these forms are quite similar and differential diagnosis is almost impossible. According to literature data, these tumours may appear as single or multiple nodules, variable in dimensions (localised MM, adenomatoid tumour, calcifying tumour of the pleura, desmoplastic small round cell tumour of the pleura), whereas in well-differentiated papillary MM and epithelioid haemangioendothelioma, no real masses are detectable, and in these two histological subtypes the pleural effusion represents the typical radiological finding. Calcifications are typical in calcifying tumour of the pleura and may be seen also in well-differentiated papillary MM. Pleural thickening is common in localised MM and epithelioid haemangioendothelioma, rare or completely absent in the other forms [41].

In all these neoplasms, surgical resection often represents the best therapeutic option; however, in the most aggressive and malignant forms it cannot be suitable or sufficient, and it can be associated with radiotherapy and chemotherapy on the basis of different therapeutic protocols, although there is no strong evidence in literature of the benefit of using a combination of these therapeutic options, due to the scant number of cases and limited information.

## Conclusions

MM is not the only existing pleural tumour, although it is the most frequent. There are other histological types that can mimic the MM by symptoms and clinical or pathological aspects. The knowledge of all possible existing pleural tumour histotypes is important to perform an adequate differential diagnosis, provide a more precise prognosis and correct management of the patient.
